# Nucleic Acid Aptamers as Potential Therapeutic and Diagnostic Agents for Lymphoma

**DOI:** 10.4236/jct.2013.44099

**Published:** 2013-06-01

**Authors:** Ka-To Shum, Jiehua Zhou, John J. Rossi

**Affiliations:** 1 Department of Molecular and Cellular Biology, Beckman Research Institute of City of Hope, Duarte, CA, USA; 2 Irell and Manella Graduate School of Biological Sciences, Beckman Research Institute of City of Hope, Duarte, CA, USA.

**Keywords:** Lymphoma, Nucleic Acid Aptamer, SELEX, Cell-Type Specific Drug Delivery, Biosensor, Nanotechnology

## Abstract

Lymphomas are cancers that arise from white blood cells and usually present as solid tumors. Treatment of lymphoma often involves chemotherapy, and can also include radiotherapy and/or bone marrow transplantation. There is an un-questioned need for more effective therapies and diagnostic tool for lymphoma. Aptamers are single stranded DNA or RNA oligonucleotides whose three-dimensional structures are dictated by their sequences. The immense diversity in function and structure of nucleic acids enable numerous aptamers to be generated through an iterative *in vitro* selection technique known as Systematic Evolution of Ligands by EXponential enrichment (SELEX). Aptamers have several biochemical properties that make them attractive tools for use as potential diagnostic and pharmacologic agents. Isolated aptamers may directly inhibit the function of target proteins, or they can also be formulated for use as delivery agents for other therapeutic or imaging cargoes. More complex aptamer identification methods, using whole cancer cells (Cell-SELEX), may identify novel targets and aptamers to affect them. This review focuses on recent advances in the use of nucleic acid aptamers as diagnostic and therapeutic agents and as targeted delivery carriers that are relevant to lymphoma. Some representative examples are also discussed.

## 1. Introduction

Lymphoma describes a group of blood cancers that develops in the lymphatic system and arise from affecting lymphocytes. In United States, more than 21,000 new cases of lymphoma are identified annually, affecting people of all ages [1,2]. Lymphoma is broadly used to describe at least 35 types of lymphomas, which are grouped based on cell histology, genetic mutations, expression of certain genes and many other characteristics [3]. Although there are many types of lymphoma, Hodg-kin’s lymphoma and non-Hodgkin’s lymphoma are two major categories of lymphomas [4–6]. Lymphoma can originate from a variety of causes, but most lymphomas are curable if caught during the earliest stage of the disease [1]. Untreated or advanced lymphoma leads to an uncontrolled proliferation of lymphocytes that spreads throughout the lymphatic system.

Aptamer technology has emerged as a valuable tool for detecting, imaging, diagnosing and treating cancer [7–10]. Aptamers are short oligonucleotides that are selected from large combinatorial pools and can bind extraordinary tightly to and effectively inhibit protein targets [11]. Aptamers can be evolved and manipulated with simplicity, but have versatility in structure and function similar to that of proteins. Since the inception of aptamer technology two decades ago, aptamers are now regularly considered along with small molecules and antibodies as potential diagnostic probes, pharmaceuticals and targeted delivery vehicles for cancer diagnosis and treatment [10, 12,13]. Regarded as “chemical antibodies”, aptamers have many attributes that are advantageous in comparison with monoclonal antibodies and small molecules. These attributes include higher affinity and specificity, greater ease of manufacture and modification *in vitro*, and better tissue penetration [14,15]. United States Food and Drug Administration (FDA) approval of the first aptamer drug (Macugen) for the treatment of age-related macular degeneration has proven to be a milestone in the applications of aptamer technology [16,17]. Many aptamers against cancer-related proteins or even intact cancer cells have been tested in clinical trials [18,19]. In addition, use of aptamers to target membrane receptors and their adoption as delivery carriers has progressed rapidly [20,21]. In this review, we highlight the importance of aptamers in lymphoma research. We discuss strategies to identify potent cancer-targeting aptamers and focus on recent advances in the use of nucleic acid aptamers as diagnostics, therapeutics and targeted delivery vehicles that are relevant to lymphoma. Some representative examples are also discussed.

## 2. The Overview of Systematic Evolution of Ligands by Exponential Enrichment (SELEX)

**Figure 1:**
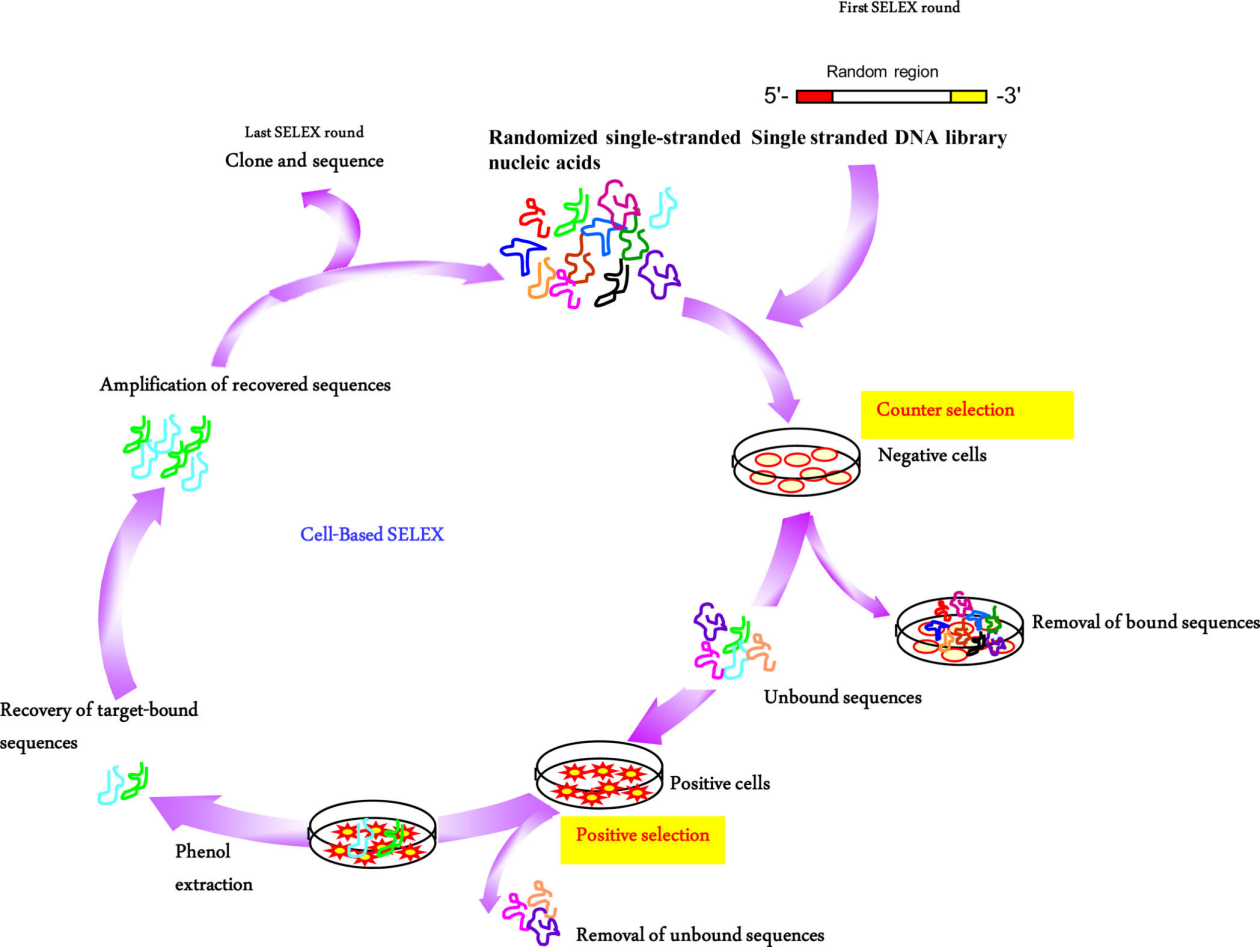
Cell-SELEX to identify aptamers that targets membrane proteins. First, a DNA library is transcribed and incubated with normal cells. Second, unbound nucleic acids are exposed to target cells that overexpress the membrane protein of interest for selection. Third, bound nucleic acids are recovered and amplified by PCR and subjected to further rounds of selection. This SELEX cycle is repeated 15 – 20 times to enrich for sequences that bind to the target cells.

Aptamer sequences that have defined and unique properties are identified from a large pool comprising a randomized combinatorial library through an iterative process of nucleic acid selection and amplification called Systematic Evolution of Ligands by EXponential Enrichment (SELEX). SELEX, which mimics Darwinian selection, was developed independently by three research groups of Gerald Joyce, Jack Szostak and Larry Gold in 1990 to describe the *in vitro* evolution of binding partners (aptamers) capable of binding to proteins, peptides, nucleic acids, small molecules, and even large organisms, such as viruses, bacteria and cells [11,22]. Typically, the SELEX process is characterized by the iteration of four essential steps: 1) binding to the target, 2) partition of target-bound aptamers, 3) recovery of target bound aptamers, and 4) amplification of recovered aptamers [21] (Figure 1).

### 2.1. Design and Construction of an Oligonucleotide Library

The starting material for the SELEX process is the synthesis of a random oligonucleotide library by standard solid phase methodology. The random single-stranded DNA consists of 20 – 80 nucleotides, flanked by a region of known sequence that can be recognized by primers in a PCR reaction. If RNA selection is to take place, a T7 RNA polymerase promoter is incorporated into the forward primer to allow *in vitro* transcription. In principle, it is thought that RNA selection provides more structural diversity than DNA selection because of the presence of 2' OH and non-Watson and Crick base pairing in RNA, but there is no clear pattern of specificity and affinity related to the chemistry of aptamers observed today [23]. Random base incorporation is achieved by using an almost equimolar mix of the four phosphoramidite precursors during the random sequence of the synthesis. Because of the varied coupling efficiencies of different nucleobases, the concentrations of phosphoramidite precursors may need to be adjusted accordingly (e.g. a ratio of A:C:G:U/T = 1.5:1.25:1.15:1.0) so there is an equal chance of each nucleotide being at any precision and no bias is introduced during this step [24].

Another issue is the length of the randomized sequence that determines the complexity of the library and its molecular diversity. If the random region is short (~10 nucleotides), then every permutation can be synthesized and the entire sequence space explored. However, such a short sequence is not sufficient for many single-stranded nucleic acid structures. The length of the oligonucleotide is also limited by the difficulty of producing longer oligonucleotide sequences by standard DNA synthesis methods. Because many natural nucleic acid-protein recognition sites comprise 15 – 25 nucleotides, a library containing 25 random nucleotides is generally considered appropriate [25]. For a randomized single-stranded nucleic acids consisting of four bases, the number of possible sequences scale at 4^n^, where n is the number of randomized base position. Typically the starting number of individual molecules used is 10^13^ – 10^15^. For a 25-mer library, there are 4^n^ = 10^15^ individual sequences in the pool, which reach the practically possible limit of sequence diversity.

After the initial DNA strand is synthesized, a primer is annealed onto the known region and the complementary strand is synthesized using the Klenow fragment of DNA polymerase I. In an RNA selection experiment, RNA is transcribed by T7 RNA polymerase using the promoter that was incorporated during synthesis of oligonucleotides. Modified nucleotides are often used instead of the natural ones to confer extra stability to the RNA molecules produced and to maintain the defined functions in the absence of toxicity [26]. For example, some popular modifications of aptamers are derivatives of the 2' ribose, such as 2' fluoro, 2' amino-methyl and 2' O-methyl derivatives [26]. Recently, two classes of modified nucleotides, Locked Nucleic Acids and spiegelmers (mirror image of aptamers) have been adapted into the PCR amplification and T7 transcription [27,28]. Thus, aptamers can be tailored to achieve certain functions through site-specific chemical modifications that are especially important for *in vivo* uses [29].

### 2.2. Partitioning and the Iterative Cycle of SELEX

The critical experimental challenge of SELEX is to design selection processes that can distinguish those molecules that are able to perform the required task, such as binding, from those that cannot, a process known as petitioning. Many powerful tools have been developed to fractionate target bound from unbound species, including centrifugation, gel shift assay, affinity chromatography, co-immunoprecipitation, surface plasmon resonance, flow cytometry, capillary electrophoresis, microfluidic devices and nitrocellulose filter binding [30–33]. As an example, in the filter binding assay, the target could be immobilized on a filter and affinity chromatography could be used; aptamers that bind to the target would be retained on the filter, while those that do not would pass through. Partitioning is the most important variable for a SELEX experiment because it can greatly simplify the processes, thereby reducing the number of selection cycles and accelerating identification of potent aptamers [22]. In general, approximate 12 iterative cycles of SE-LEX are sufficient to achieve significant enrichment within the library of an aptamer that has high binding affinity with the target. The inherent properties of selected oligonucleotides can be further fine-tuned for different purposes by adjusting the stringency parameters, such as salt concentration and number of washes.

### 2.3. Generation of Cell-Specific Aptamers

To date, a considerable number of aptamers have been evolved to target a specific cell type or subpopulation of cancer cells through either protein-based or cell-based SELEX [18,19]. The traditional approach requires a purified soluble form of the target proteins in desirable concentrations. However, this method has inherent limitations because the purified recombinant protein may not fold into a similar conformation as under physiological conditions. Moreover, the native protein may be present in a modified form or exist as a protein complex that may be masked and therefore inaccessible to the aptamers [34]. This problem is especially common when the trans-membrane domain of a receptor embedded in the plasma membrane is not exposed to aptamer binding. To overcome this, research groups have modified the traditional SELEX protocol to enable use of whole living cells as targets (Cell-SELEX).

The advantages of Cell-SELEX include being able to select native aptamers without going through the processes of protein expression and purification [35]. In addition, Cell-SELEX can be performed when unknown targets are expressed on the cell surface (Blind Cell-SELEX) as this strategy relies on the differences between two distinct populations of cells (target diseased cells versus control healthy cells) that have particular defined features, such as protein expression levels and phenotypes [36–38]. In theory, aptamers isolated from cell-SELEX will only recognize the target cells, but not control cells. Akin to traditional protein-based SELEX, Cell-SELEX is also characterized by the iterative cycles of the four main steps described above, albeit using living cells instead of purified proteins (Figure 1). Because intact living cells contain many native receptor proteins, a counter-selection step is required to avoid non-specific binders. For example, if the selection step involves a cell line that overexpresses a cell surface receptor of interest, the counter-selection step could involve a related cell line that lacks or has a very low expression of the target protein. Generally, Cell-SELEX requires approximately 15 −20 cycles of selection, which is more than that of protein-SELEX [7]. Because many surface complexes are present on cells and these complexes consist of multiple proteins, multiple families of aptamers are expected to be identified, and the selected aptamers must be carefully characterized [39]. Moreover, Cell-SELEX does not discriminate between living and dead cells. Because dead cells have reduced membrane integrity and can non-specifically internalize nucleic acids in a sequence-independent manner, cells should be kept healthy during selection as any damages to the cells may incur risk of selection failure [7,21]. Because Cell-SELEX facilitates the development of and simplifies the identification of molecular probes for recognizing cancer cells, this technology opens the way to an improved understanding of cancer development and provides a platform for developing new tools for the diagnosis and treatment of cancer (Table 1).

## 3. Aptamers as a Potential “Next-Generation” of Diagnostic Tools

Lymphoma is fatal if left untreated or diagnosed at a late stage. Some classes of lymphoma, such as Burkitt’s lymphoma, are particularly acute and aggressive and therefore require rapid and accurate diagnosis and prognosis to aid in prevention, therapy and detection of minimal residual diseases [40]. There are a battery of options to diagnose lymphoma in the clinic, involving a combination of technologies, such as immunophenotypic analyses using flow cytometry or microarray, karyotyping analysis of peripheral blood and bone marrow, and detection of malignant cell mutations through PCR [41–43]. These methods require relatively simple technical skills and have a short turnaround time, but impose certain limitations. For example, immunophenotyping by flow cytometry requires the use of multiple monoclonal antibodies to reliably detect lymphoma, thereby complicating the diagnosis and increasing the cost of the method [44, 45]. Although PCR can sensitively examine the RNA expression of certain genes to detect cancerous cells, occult or early-stage tumors cells have very low signal levels that could result in a false-negative misdiagnosis [46]. Therefore, development of new diagnostic tests for sensitive, accurate and economical identification of lymphoma cells remains an unmet medical need.

Nucleic acid aptamers have several parallels with antibody technology, but also have many advantages over antibodies as tools for molecular recognition [47,48]. The most significant advantage is that aptamers are more tolerant of chemical and temperature changes and have longer shelf lives than antibodies, because they are stabilized nucleic acids. Other advantages include ease and reproducibility of synthesis, reducing both manufacturing costs and batch to batch variability, which are intrinsic problems with monoclonal antibody-based diagnostic kits. As small, nonimmunogenic probes, aptamers may have better tissue penetration and cellular uptake and a higher ratio of target accumulation, thereby affording great potential for *in vivo* use. Several aptamers are being used to lay the groundwork for new point-of-care diagnostic approaches for leukemia and lymphoma.

### 3.1. Anti-CD30 Aptamers

CD30 antigen is a transmembrane protein receptor of the tumor necrosis factor (TNF) family that is normally expressed in activated T and B cells, but is overexpressed in tumor cells of anaplastic large cell lymphoma (ALCL) and Hodgkin’s lymphoma [49,50]. Thus, expression of CD30 on tumor cells is an important and unique bio-marker for diagnosis of these types of lymphomas and for differentiating them from other tumors. Anti-CD30 antibodies have been routinely used in the clinic to detect CD30 expression [51]. Anti-CD30 RNA aptamers have also been explored for diagnosis of these lymphomas, and were identified using the extracellular domain of the receptor activator of nuclear factor-ĸB (RANK) as an evolution target. Interestingly, these aptamers demonstrated strong and specific binding to CD30 proteins, with an affinity (K_D_) more than 1000 times higher than that to other proteins in the TNF family [52].

The use of these anti-CD30 aptamers as probes to detect lymphoma cells was explored using flow cytometry and immunostaining [53,54]. In the flow cytometry approach, the anti-CD30 aptamers were fluorescently labeled and tested with cultured ALCL and Hodgkin’s lymphoma [53]. Both flow cytometry and fluorescence microscopy experiments demonstrated specific binding of the aptamers to these lymphoma cells at a sub-nanomolar scale and CD30 aptamer and the CD30 antibody recognized the same sets of cells among various cultured tumor or healthy cell lines and had identical specificity and sensitivity [53]. Molecular studies also showed that both anti-CD30 aptamers and antibodies independently bound to the CD30 proteins, suggesting that the CD30 aptamer probe could serve as a replacement and/or supplement for the CD30 antibodies in multicolor cell immunophenotyping analysis [53]. Similarly, the same anti-CD30 aptamers were used as probes for immunostaining of paraffin-embedded and formalin-fixed lymphoma tissues. Expression of CD30 on the lymphoma cells was recognized by biotinylated anti-CD30 aptamers, which were subsequently visualized by horseradish-peroxidase color development [54]. In this assay, the aptamer approach exhibited superior diagnostic values to the antibody approach in that the anti-CD30 aptamers could specifically stain the lymphoma cells and required a shorter incubation time and lower temperature for antigen retrieval [54].

### 3.2. TD05 Aptamers

Burkitt’s lymphoma is an acute blood cell cancer and one of the most aggressive of all human cancers. Because Burkitt’s lymphoma spreads so quickly, early and accurate diagnosis is essential for treating its victims. Burkitt’s lymphoma is typically diagnosed through morphological inspection of the blood cells and biopsied tissue samples, but this approach is rather time-consuming and ineffective [55]. Because aptamers represent great options in molecular recognition and understanding of diseases at the molecular level, TD05 aptamers have been designed to diagnose Burkitt’s lymphoma. TD05 aptamers were evolved by applying Cell-SELEX against a Burkitt’s lymphoma cell line, Ramos cells [56]. Initially, a panel of DNA aptamers was selected for Ramos cells. These aptamers had strong binding affinity and some exhibited distinguished specificity. The TD05 aptamers were one of these aptamers, but they only recognized Ramos cells and did not bind to normal CD19^+^ B cells or other bone marrow cells [56]. Subsequent proteomics experiments determined that an immunoglobulin heavy mu chain, which is a B-cell antigen receptor expressed on the surface of Burkitt’s lymphoma cells, was a binding target for the TD05 aptamers [57].

**Figure 2:**
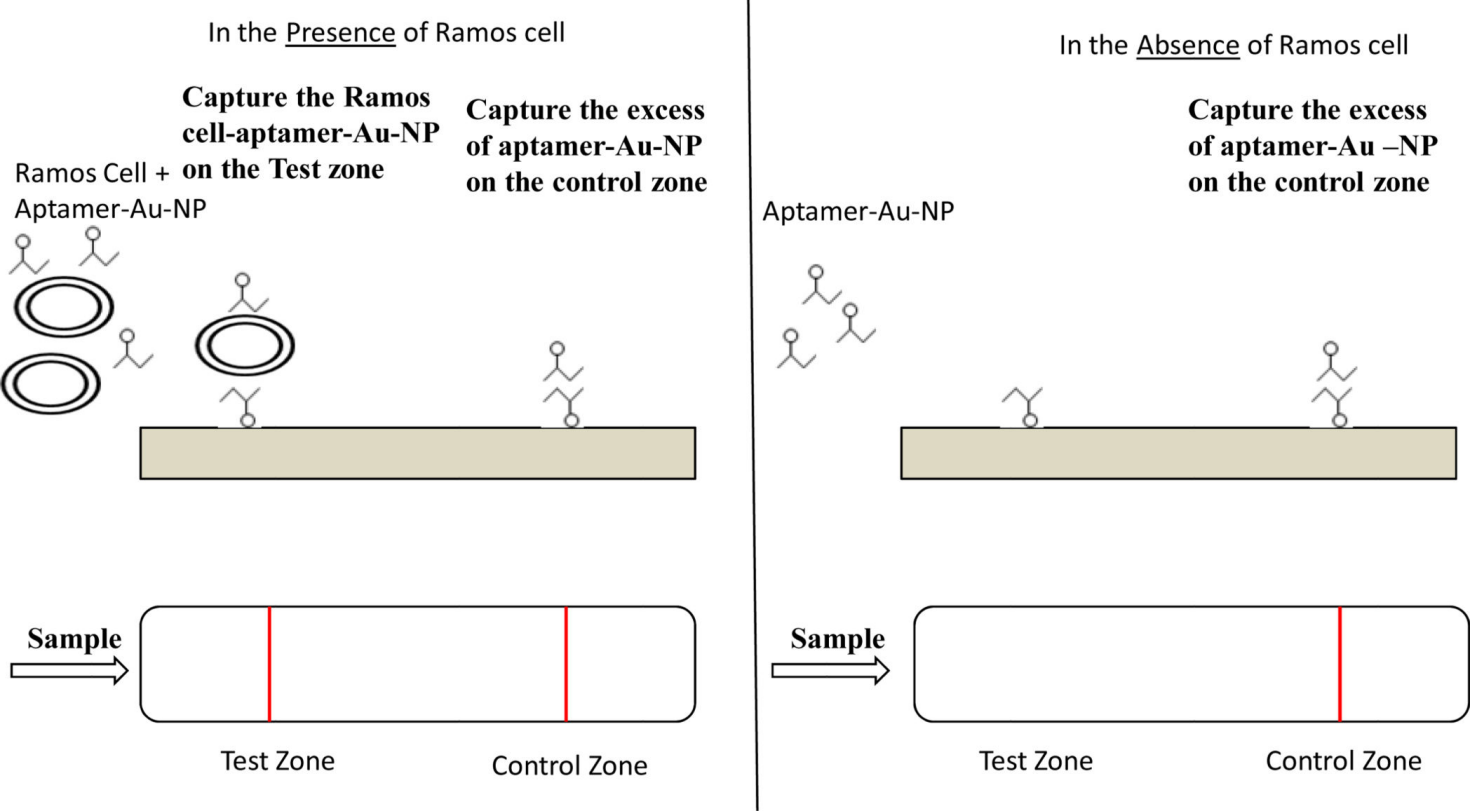
A TD05 aptamer-nanoparticle strip biosensor for detecting a Burkitt’s lymphoma cell line (Ramos cells). A sample solution that contains Ramos cells is recognized by TD05 aptamer gold nanoparticles (TD05-Au-NP). The TD05 Au-NP-cell complexes are captured on the test zone by a second binding event between Ramos cells and immobilized Ramos cells that recognize aptamers (TE02). The accumulation of Au-NP results in a red band. The excess Au-NP continues to migrate and is captured on the control zone by a hybridization reaction between TD05-Au-NP and an oligonucleotide complementary to the TD05 aptamers, leading to a second red band. In the absence of Ramos cells, only the red band in the control zone is observed, showing that the assay is working properly.

Because of their specificity and affinity to Ramos cells, TD05 aptamers have been widely reported for use as a diagnostic [58,59]. One significant step forward enabled by aptamers has been the development of a simple, sensitive dipstick test for lateral flow-based detection of Burkitt’s lymphoma cells, using an aptamer-nanoparticle strip biosensor (Figure 2) [60]. This approach uses technology similar to that in commercially available pregnancy test kits, and provides a litmus-test type of assay. In the original report of this approach, gold nanoparticle aggregates that were responsive to Ramos cells were designed in which the thiolated TD05 aptamers were immobilized on the gold nanoparticles, while another Ramos cell selective aptamer (TE02), which was identified along with the TD05 aptamers in Cell-SELEX, was immobilized on the test zone of the strip [60]. The principle of the assay was that the sample solution or human blood sample would migrate by capillary action along the strip. In the presence of Ramos cells, the TD05 aptamers immobilized on the gold nanoparticles would interact with the cells to form the gold nanoparticles aptamer-cell complex and continued to migrate along the strip. The gold nanoparticle-aptamer-cell complexes were captured at the test zone by a second aptamer, TE02, immobilized on the strip. Because of the optical features of gold nanoparticles, the accumulation of gold nanoparticle-aptamer-cell complexes at the test zone produced a red line. Excessive amounts of gold nanoparticle-aptamer conjugates continued migrating to the control zone, in which the gold nanoparticle-aptamer complex was captured by a DNA sequence complementary to the TD05 aptamers, thereby forming a second red line to show the biosensor was working properly. Whereas, if the sample did not contain Ramos cells, only one red line was expected in the control zone and no red band would be seen in the test zone. Optimally, this biosensor could detecting a minimum of 4000 Ramos cells, thus providing a rapid, sensitive and economical tool for the detecting Burkitt’s lymphoma cells.

Alternatively, the TD05 aptamers were used as molecular probes for real time *in vivo* tumor-target imaging inside live animals [58]. The TD05 aptamers were labeled with fluorescent dyes, such as Cy5, and then injected into mice that were engrafted with Ramos cells. *In vivo* fluorescence imaging experiments showed that only the Cy-5-labeled TD05 aptamers accumulated in the tumor tissues, while non-specific control probes were rapidly cleared from the body. Notably, the probe produced very high signal-to-background ratios during imaging and could distinguish Ramos cells from other circulating tumor cells, such as T-cell acute lymphoblastic leukemia (ALL) cells and CCRF-CEM *in vivo*. However, because nucleic acid aptamers have serum degradation issues in biological fluids, a number of chemical modifications have been introduced to various parts of aptamer structures to increase the feasibility of using aptamers *in vivo* (e.g. nuclease resistance and conformational stability). One notable modification for *in vivo* cancer imaging is the design of polyethylenimine (PEI)-aptamer probes [59]. When TD05 aptamers are immobilized onto PEI-polymers, the degradation rate of the aptamers is greatly reduced. A whole animal fluorescence imaging study demonstrated that the fluorescence signal from tumor tissues that were imaged by fluorescently labeled PEI/TD05 aptamer nanoparticles remained elevated for five hours post-injection, while the signal when naked TD05 aptamers was used was only stayed for 45 minutes. This interesting strategy can significantly enhance the pharmacokinetics and half-life of aptamers, which is important for offering a feasible and versatile method to image tumors *in vivo* with high specificity and sensitivity.

### 3.3. Sgc8 Aptamers

Sgc8 aptamers are DNA aptamers that were generated through a cell-based selection strategy for the specific recognition of leukemia cells [61]. In the selection process, two tumor cell lines were selected as ligands. An ALL cell line, CCRF-CEM, was chosen as the target for selection, while a Ramos cell line was used as a counter selection to minimize the DNA sequences that could bind to common ligands on the surface of both hematopoietic cell lines [61]. The Sgc8 aptamers were one of the enriched sequences that could specifically recognize cancer cells from T cell ALL patients and cultured CCRF-CEM cells (K_D_ = 800 pM), but did not bind to normal CD3^+^ T cells or any other bone marrow cells [61]. Subsequent studies using affinity chromatography and mass spectroscopy discovered that protein tyrosine kinase 7 (PTK7) is a molecular target of Sgc8 aptamers [62]. PTK7, also known as colon carcinoma kinase-4, is a transmembrane receptor that is expressed in normal hematopoietic cells, but its expression is elevated in all T-ALL cell lines [63]. Given the specificity and affinity of Sgc8 aptamers, several innovative technologies have been developed to exploit the diagnostic potentials of this aptamer and are discussed below. Although these technologies used the Sgc8 aptamers to recognize T-cell ALL, they can be, in general, applied to lymphoma or blood circulating tumor cells.

The simplest practical way to visualize tumor *in vivo* relies on a strategy that uses reporter-bearing probes in which the cell-recognizing ligands are labeled with reporter molecules, such as fluorescent dye, and then bind to target tumor cells. Accumulation of the reporter ligands around cells then leads to an increased signal as compared to the surrounding environment. However, because these molecules usually have a constant signal, the image contrast is critically hindered by high background. To address this complication, an activable aptamer reporter probe based on a cell membrane protein-triggered conformation change was designed [64]. The design of the activable aptamer reporter probe consists of three elements: 1) a tumor-targeting aptamer e.g. Sgc8 aptamers, 2) a poly T linker and 3) a short DNA sequence complementary to part of the aptamer that has a fluorophore and a quencher linked at either terminus [64]. In the absence of target proteins, the probe is in an inactive form in which the short DNA sequence is hybridized with the aptamer. This keeps the fluorophore and the quencher in close proximity, leading to quenched fluorescence. Conversely, when the target cancer cells are present, the conformation of the aptamer probe is spontaneously reorganized on binding, causing the fluorophore to separate from quencher. Accordingly, a strong fluorescence signal is generated in response to the interaction of the probe with the target cells. *In vitro* analysis and *in vivo* imaging of CCRF-CEM cells were performed by incorporating the Sgc8 aptamers into the probe. This demonstrated that the Sgc8 aptamer probe exhibited enhanced background contrast and desirable specificity in differentiating CCRF-CEM tumor from other blood tumors and normal cells [64]. This sensitive technology is expected to facilitate an early detection of cancer cells and significantly shorten the diagnosis time.

Detecting cancer during its earliest stages or prior to metastatic relapses is difficult and sometimes requires multiple complicated diagnostics and lab tests to provide conclusive results. A common detection method involves spotting out live tumor cells from body fluids, such as blood. However, the cancerous cells are usually present at very low concentration that is below the sensitivity limit of conventional methods. Therefore, developing a non-invasive, fast and inexpensive method for enriching and detecting cancerous cells is critically important for early cancer diagnosis. Recently, an aptamer-based mi-cofluidic device was reported that demonstrated an exceptional ability to enrich for scarce cancer cells in a background of healthy cells on the basis of molecular interactions [65,66]. In this case, CCRF-CEM targeting Sgc8 aptamers were first biotinylated and covalently immobilized on a microfluidic channel that had surface-bound. The cell mixture was then pumped into the channel [66]. Because the Sgc8 aptamers are specific to CCRF-CEM cells, target cells in the cell mixture were captured and enriched. These cells were subsequently visualized by optical microscopy to measure the cell-surface density. Generally, this device obtained 80% capture efficiency and 97% purity [66].

In a further study of this method, the same research group extended the design of the microfluidic device to allow simultaneous sorting, enrichment and detection of three different types of cancer cells from a complex sample by using three leukemia- or lymphoma-specific aptamers (Sgc8 aptamers for CCRF-CEM cells, TD05 aptamers for Ramos cells and Sgd5 aptamers for Toledo cells [non-Hodgkin’s B cell lymphoma cell line]) [65]. These aptamers were immobilized on different regions within the microfluidic channel and were used to capture and enrich target cells through molecular interactions. This approach achieved a 135-fold enrichment of sorted cancer cells with ~96% purity [65]. Notably, this simple assay did not require any pre-treatment of cells and could be completed within minutes. However, alternative methods for detecting captured cells should be considered because it is not practical to scan the entire microfludic device with a microscope.

Another approach could involve combining aptamers with nanotechnology. Nanotechnology involves the creation and application of nanometer-scale materials that have a wide variety of elegantly patterned array and high-ordered structures. These nanomaterials perform diverse biological functions and their novel designs have inspired biomimetic strategies. In particular, nanomaterials can provide a large surface area as multivalent ligand scaffolds that allow the incorporation of multiple recognition ligands, such as aptamers [67]. Au-Ag nanorods are one promising nanomaterial that has been used as a scaffold for multivalent binding by aptamers on the rod for use in detecting cancer cells. These nanorods have been fabricated with up to 80 fluorescently labeled Sgc8 aptamers through thiol linkages and displayed much stronger fluorescence signal than that of the original aptamer probe [68]. Additionally, the attachment of multiple aptamers on the nanorod surfaces increased the binding affinity by at least 26-fold as compared to the individual Sgc8 aptamer alone. When incubated with target CCRF-CEM cells, the nanorod-Sgc8 aptamer showed significantly enhanced fluorescence signal as measured by flow cytometry [68]. Although the use of nanorods has not been clinically tested, this is an excellent example of using nanomaterials with desirable properties to perform cellular imaging.

## 4. Therapeutic Applications of Aptamers in Lymphoma

The initial therapeutic approach chosen for a patient is based on their specific form of lymphoma and stage of disease [69]. In general, chemotherapy and radiation therapy are the two major treatments of choices. Chemotherapy uses small molecule or monoclonal antibody-based drugs, such as the CHOP regimen, to kill cancer cells, while radiation therapy uses high-energy X-rays to shrink tumors. Although both approaches are relatively effective in adults with leukemia and lymphoma, the therapies are ineffective in elderly patients because of their ability to survive in the harsh treatment. Thus, only a small percentage of adult patients enjoy long-term disease free survival. Additionally, these therapies are not target-specific, so they do not just kill cancer cells, but also affect healthy non-cancerous cells, which can lead to harmful side-effects. In some forms of lymphoma, such as non-Hodgkin’s lymphoma, hematopoietic stem cell transplantation may be a potentially curative option, but this approach is limited by the high risk of surgery and the availability of a matched donor [69]. Therefore, there are still significant opportunities for developing alternative therapies against lymphoma.

The major goal of cancer therapeutics continues to be the complete eradication of tumor cells without inducing toxicity to the patient’s normal tissues. Over the past decade, aptamer-based therapeutics have emerged as promising alternatives to chemotherapy and radiotherapy because of their ability to bind to and specifically inhibit targets, while causing minimal or no adverse side-effects [18]. In particular, the chemical nature of aptamers makes them attractive potential therapeutic agents that could rival small molecules and monoclonal antibodies. Akin to traditional small molecule drugs, such as Viagra (Pfizer) and Gleevec (Novartis), aptamers fit into crevices on protein surfaces, especially the active sites of enzymes to inhibit their catalytic activity. Aptamers can also form clefts that bind protruding parts of protein [70]. This increased surface area of contact with targets, allowing aptamers to bind more specifically and tightly, thereby disrupting protein-protein or even cell-cell interactions more effectively than small molecule or monoclonal antibody based drugs [71]. This is particularly important in the development of anti-cancer drugs as the signaling cascades triggered by cancer therapeutics are initiated from ligand-receptor interactions. Typically, small molecule inhibitors are generally not suitable, as preferably a molecule with a large surface area to inhibit macromolecular interactions is more desirable. Therefore, most attempts to develop small molecule drug pursue enzymes that have small active sites, such as kinases and phosphatases. But receptor proteins or nuclear proteins are ideal candidates for drug therapy in many fields, including cancer and degenerative diseases, as surface receptors tends to be overactive in these diseases. Thus, aptamers, with their large surface binding areas and low dissociation constants when bound to targets, are ideal for inhibiting ligand-receptor complexes in cancer therapies.

Aptamer therapeutics are becoming more established now that an aptamer (Macugen, Pfizer) targeting VEGF for the treatment of macular degeneration is in clinical use [16] and several other aptamers for treating AML, renal cell carcinoma and non-small cell lung cancers are in various stages of the clinical development pipeline [10, 70]. Moreover, aptameric drugs are classified as chemical drugs rather than biological entities, which will facilitate FDA approval.

### 4.1. AS1411 Aptamers

AS1411 is a 26-nt G-quadruplex DNA aptamer that has just completed Phase II clinical trials as a first anticancer nucleic acid agent, for the treatment of renal cell carcinoma and AML [12]. The discovery of AS1411 was somewhat coincidental—a result of using the “purine motif” approach of using triplex-forming oligodeoxynucleotides to regulate gene expression [12,72]. This results in DNA sequences that consist entirely of thymine (T) and guanine (G). One of these sequences was a 26-mer that became AGRO100, while being developed at Aptamera. AGRO100 was renamed as AS1411 after it was acquired by Antisoma. In 2011, AS1411 was acquired by Advanced Cancer Therapeutics (ACT), and renamed ACT-GRO-777.

AS1411 binds to both nucleolin and to nuclear factor-*k*B essential modulator (NEMO), which is involved in activating the critical transcription factor NK-*k*B [73]. Nucleolin is a multifunctional protein that has diverse roles in cell growth and death. One important feature of nucleolin is as a shuttle protein that transports from the plasma membrane to cytoplasm and nucleus. Nucleolin is overexpressed in many cancer cells, including AML, and breast, lung, and pancreatic cancer [74–78]. A recent study also showed that nucleolin modulates Interleukin-9 (Il9) expression in T-cell lymphoma cells [79]. Another major role of nucleolin is to bind to an AU-rich element in the 3’UTR of Bcl2 mRNA [80]. This binding protects the anti-apoptotic Bcl-2 from degradation, resulting in stabilization of Bcl2 mRNA, which subsequently promotes the cancer cells to overproduce Bcl2 and avoid apoptosis [80]. Early preclinical studies demonstrated that micromolar concentrations of AS1411 could inhibit proliferation of a wide variety of tumor cell lines, and suppressed tumor growth in a xenograft animal model [78]. Phase I clinical trials (involving 30 patients) were completed in 2006 and indicated that AS1411 was safe and well-tolerated [72]. Phase II trials for AML are showing positive interim results (ClinicalTrials.gov Identifier: NCT00512083 and NCT01034410). Recent research has explored the use of AS1411 as a carrier for the delivery of cancer drugs specifically into cancer cells by nucleolin mediated internalization [81].

### 4.2. NOX-A12 Spiegelmers

NOX-A12 is a 45-nucleotide long spiegelmer aptamer that was developed by a proprietary spiegelmer technology^®^ in Noxxon Pharma (Germany). Spiegelmers are mirror image (L-steroisomer) RNA aptamers. An important feature of spiegelmers is that they have tremendous stability in the body because nucleases recognize natural D-nucleic acids, but not L-nucleic acids (Spiegelmers) [82]. Additionally, spiegelmers possess all other characteristics of canonical aptamers, such as high binding affinity and selectivity to the target, passive immunogenicity and ease of manufacture [82].

The identification of NOX-A12 spiegelmers combined screening of large combinatorial RNA libraries with a chemical mirror technology in which NOX-A12 was evolved from the natural D-RNA library by using a mirror image target (D-amino acid), and then was synthesized as L-RNA to bind the natural target (L-amino acid) [82,83]. NOX-A12 spiegelmers specifically antagonize CXC chemokine ligand 12/stromal cell derived factor-1 (CXCL12/SDF-1), which is a key regulatory chemokine for migration of leukemia stem cells to the bone marrow [84]. CXCL12 also plays an important role in tumor growth and metastasis by binding with high affinity to two chemokine receptors, CXCR5 and CXCR7 [84]. Both of these receptors appear to contribute to metastasis of cancer cells to regions with elevated CXCL12 expression, such as lymph nodes and bone marrow. Moreover, CXCL12 signaling has been shown to play a major role in the pathophysiology of chronic lymphocytic leukemia (CLL), especially in the interaction of leukemic cells with the tissue microenvironment [85]. Thus, inhibition of CXCL12 binding to its receptors by NOX-A12 spiegelmers has the potential to interfere with the CXCL12 anchor and prompt leukemia stems cells to re-enter the cell cycle and become available for chemotherapeutic attack.

NOX-A12 spiegelmers have also shown promising pre-clinical anti-tumor activity in various solid and hematological tumor models. A Phase 1 clinical trial involving 48 healthy subjects was launched in 2009 and showed that NOX-A12 spiegelmers were safe and well-tolerated. Notably, the pharmacokinetic profile showed that NOX-A12 spiegelmers have a very long half-life of 37 hours. Recently, two Phase IIa clinical studies of NOX-A12 have begun for hematological oncology indications, including non-Hodgkin’s lymphoma, chronic lymphocytic leukemia and multiple myeloma (Clinical-Trials.gov Identifier: NCT01486797 and NCT-01486797). This study will involve 33 relapsed CLL patients who will receive NOX-A12 spiegelmers in combination with a background therapy of chemotherapeutic agents. Interim results are expected to be available in early 2013.

## 5. Aptamer “Smart Bomb” Mediates Delivery of Drugs to Cancer Cells

The emphasis on cancer therapeutics has now shifted to the development of tumor-specific, targeted therapies in the hope that only the cancer cells will be targeted and killed by the drugs, while non-cancerous normal cells will not be affected. Antibody drug conjugates, which couple the cell-recognizing advantage of antibodies with the cytotoxic potential of chemotherapy, herald the promise of targeted cancer therapy, and several antibody drug conjugates are currently undergoing clinical evaluation [86]. Indeed, the development of antibody drug conjugates are one of the major interests in pharmaceutical and biotechnology companies nowadays [87]. Currently, only two antibody-drug conjugates have been approved by FDA for the treatment of hematological cancers, and only one of these is still in clinical use. Gemtuzumab ozogamicin (Mylotag, Pfizer) was the first FDA-approved antibody-drug conjugates designed for patients with relapsed AML, but was discontinued because of significant side effects in a post-approval trial [87,88]. Gemtuzumab targets CD33 antigen expressed on both leukemic blast cells and normal hematopoietic cells [88]. Brentuximab vedotin (Adcetric, Seattle Genetics Inc.) was the second FDA approved antibody drug-conjugates for the treatment of Hodgkin’s Lymphoma [89]. Unlike gemtuzumab ozogamicin, which also targets normal cells, Brentuximab vedotin only targets CD30, which is an established marker for classic Hodgkin’s lymphoma, anaplastic large cell lymphoma and embryonal carcinomas [90–92]. In brentuximab vedotin, the antibody is conjugated to monomethyl auristatin E, which inhibits cell division by preventing polymerization of tubulin [90,93].

Despite the seemingly promising clinical results on the use of antibodies for targeted cancer therapy, these drugs face many challenges that limit their successful clinical translation. Some of these challenges might be due to the inherent properties of monoclonal antibodies as specific therapeutic deliverables [87]. For example, in gemtuzumab ozogamicin, only 50% of the anti-CD33 gemtuzumab antibody was attached to the chemotherapeutics (4 – 6 drug molecules per antibody), while the remaining antibody was unconjugated, suggesting that the potency of the antibody-drug conjugates was undermined [87,94]. Arguably, aptamer technology is an attractive alternative for use as drug carriers during targeted drug delivery. Aptamers have several advantages over antibodies used in targeted delivery, particularly for cancer therapy [15, 18]. First, manufacture of aptamers relies on chemical synthesis and therefore is much easier to scale-up with low batch-to-batch variation. Second, aptamers are much smaller than antibodies, which can lead to better tissue penetration and cell internalization. Third, using aptamers as anti-receptors will yield superior specificity and the lowest antibody-inducing activity as compared to protein anti-receptors, thus providing a means for repeated administration and treatment of chronic diseases, such as cancers. Last, attachment of chemotherapeutic drugs, imaging agents or any functional group can be readily computed during aptamer synthesis. Many aptamers have been identified for hematological cells and examples of aptamer-mediated delivery are discussed below.

### 5.1. TD05 Aptamer-Mediated Delivery of a Photosensitizer

The TD05 aptamers, which target the immunoglobulin heavy mu chain in a Burkitt’s lymphoma cell line, were engineered with a photosensitizer for selective photodynamic therapy of targeted tumor cells [95]. Photodynamic therapy exploits the ability of nontoxic light-sensitive compounds called photosensitizers to produce cytotoxic reactive oxygen species when illuminated and it is widely recognized as a minimally toxic and minimally invasive approach [96]. Recently, the photodynamic therapy-based approach has been used clinically to treat several medical conditions, but it is limited to locally exposed diseases, such as skin cancers and severe acne. The largest challenge of using this technology for diseases inside the body is the effective localization of the cytotoxic photosensitizers at the diseased cells. Because the TD05 aptamers can specifically recognize Ramos cells, an aptamer-photosensitizer conjugate was engineered. This conjugate contained the cytotoxic porphyrin-based photosensitizer reagent Chlorin 6 [81,95]. *In vitro* studies revealed that these aptamer-photosensitizer conjugates were highly selective to Ramos cells and effectively generated free-radicals that triggered Ramos cell upon illumination. This aptamer-photosensitizer conjugate was highly selective to Ramos cells, but did not affect acute lymphoblastic leukemia or myeloid leukemia cell lines [95]. To increase their binding affinity to the target and their half-life in the body, the TD05 aptamers were designed to attach a lipid tail at the end of the aptamers to form a self-assembled aptamer-micelle nanostructure [97]. Alternatively, a construct containing multiple TD05 aptamers also successfully improved the specificity and binding kinetics of the aptamers.

### 5.2. AS1411 Nucleolin Aptamer-Mediated Drug Delivery

The aptamer-mediated delivery approach is not limited to delivery of therapeutic cargos to cell cytoplasm, but can also extend to nuclear delivery. Nucleolin acts as a shuttle to transfer molecules between the cell surface and the nucleus [98], and several hematopoietic cancer cell lines, including AML and chronic lymphocytic leukemia/small lymphocytic lymphoma, expressed higher levels of nucleolin than normal cells [74,78]. Moreover, molecules that bind to nucleolin are then internalized by the cells. These data suggested nucleolin as an excellent candidate for targeted delivery of anti-cancer agents as a means for internalization of nucleolin-binding AS1411 aptamers.

Shieh *et al*. reported a novel strategy of using AS1411 aptamers as drug carriers to target cancer cells for photo-dynamic therapy [81]. The AS1411 aptamers were complexed with 6 photodynamic molecules, TMPyP4, to form multivalent AS1411 apt-TMPyP4 conjugates through electrostatic interactions. This approach is similar to cocktail therapy, in which a mixture of drugs is used to produce a synergistic effect. The AS1411 apt-TMPyP4 conjugates accumulated in nucleolin-expressing cells lines, likely because of nucleolin-mediated internalization *via* AS1411, and damaged these cells upon exposure of light [81]. Using a similar strategy, Aravind *et al*. reported the use of lipid-polymer hybrid nanoparticles loaded with chemotherapeutic agents and decorated with AS1411 aptamers for targeted drug delivery [99]. These lipid-polymer hybrid nanoparticles were composed of 6 elements 1) a hydrophobic polymeric core made of poly D, L lactic-co-glycolic acid (PLGA), 2) a chemotherapeutic drug, paclitaxel, 3) a fluorescent dye, 4) a lipid layer made of lecithin, 5) a PEGylated phospholipid, DSPE-PEG2000-COOH and 6) AS1411 aptamers on the surface [99]. Physical analyses confirmed that the nanoparticles were spherical and ranged in a size from 60 to 110 nm. Subsequent drug-loading studies showed that this nanoparticle had an enhanced cytotoxic effect, higher encapsulation efficiency and superior sustained drug release than corresponding non-target nanoparticles [99].

Recently, Sullenger and colleagues devised an aptamer-based conjugate that used antisense RNA oligonucleotides as cargos that were delivered to the nucleus to modulate gene expression [100]. The antisense oligonucleotides employed, known as splice-switching oligonucleotides, were single stranded modified RNA molecules that bind to a splice site or splicing enhancer to sterically hinder access to cellular splicing events, thereby biasing the splicing pattern of targeted mRNAs. The system is made entirely of nucleic acids, in which the splice-switching oligonucleotides are appended to the 3' termini of the AS1411 aptamers [100]. As nucleolin translocated from the cell surface to the nucleus, the conjugates were internalized into target cells and altered pre-mRNA splicing. This approach was effective at lower therapeutic doses than when the splice-switching oligonucleotides were used alone.

**Figure 3:**
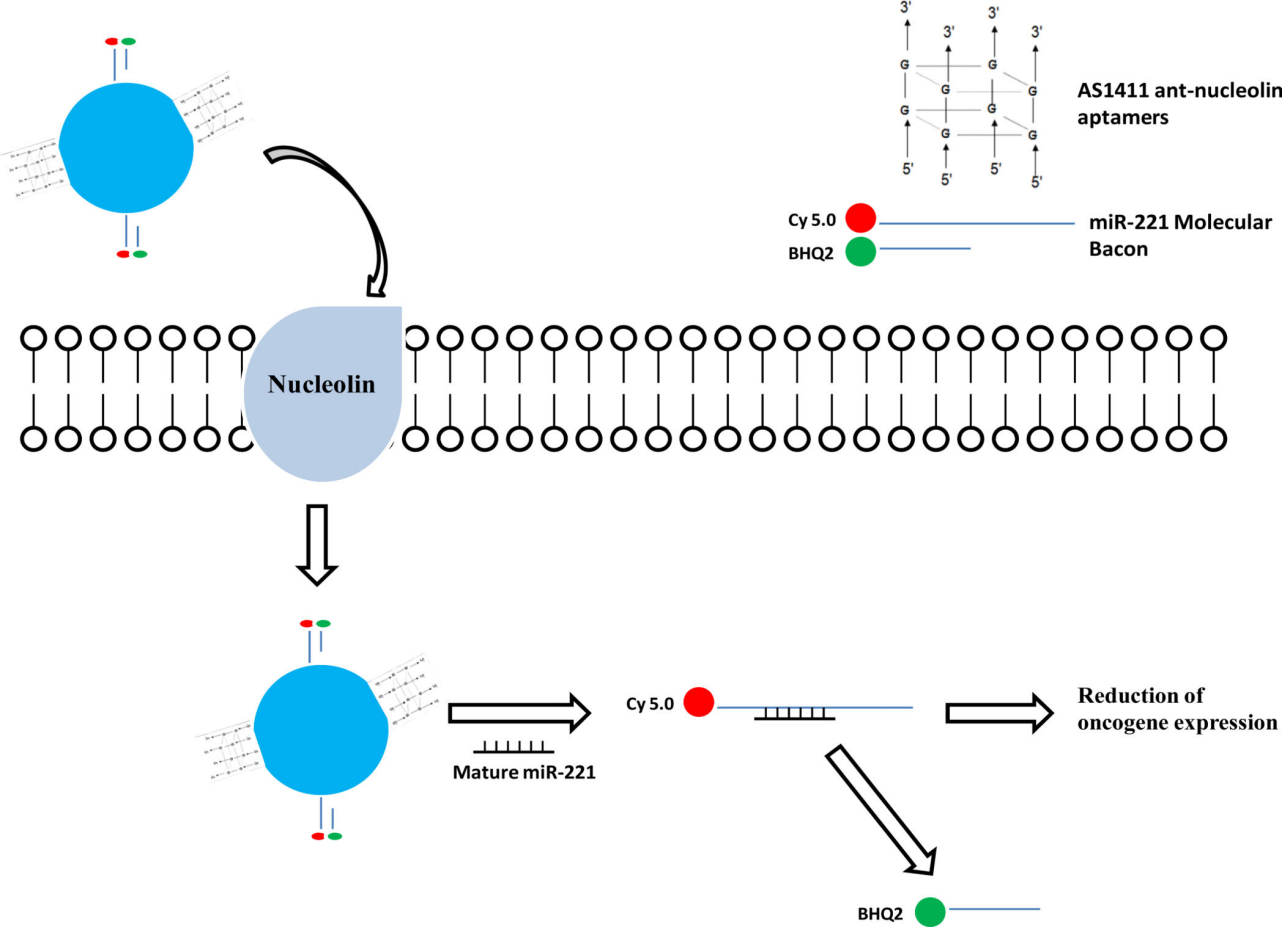
An AS1411-miR-211 molecular beacon theranostic. AS-1411 aptamers on the nanoparticles escort the theranostic to nucleolin positive-cancer cells and promote internalization of the theranostic by binding with cell surface nucleolin proteins. Once internalized, the reducing cytoplasmic environment causes miR-221 molecular beacon to be separated from the nanoparticles. The miR-221 molecular beacon then hybridizes with cellular mature miR-221, leading to separation of the double-stranded molecular beacon and producing a strong fluorescent signal. Additionally, the binding of cellular miR-221 to miR-221 molecular beacon inhibits the functions of endogenous miR-221, reducing oncogene expression. In contrast, Cy 5.0 and BHQ2 fluorescence quencher in the miR-221 molecular bacon remain in close proximity in the absence of cellular miR-221.

Kim *et al*. developed a cancer-targeting theranostic probe that can detect and inhibit cancer cells at the same time [101]. The benefit of being able to visualize therapeutic agent is that caregivers can determine the location and effectiveness of the drug’s action, providing a clear and precise diagnostic. This probe used micro-RNA as a cancer biomarker and employed a magnetic fluorescence nanoparticle conjugated with AS1411 aptamers and an miRNA-221 molecular beacon (Figure 3) to simultaneously target cancer cells, image intracellularly expressed miRNA-221 and inhibit function of cellular miRNA-221 [101]. Collectively, the resulting nanoparticles (MFAS miR-221 MB) displayed superior selectivity in nucleolin-expressing cancer cells. When incubated with target cells, the nanoparticles were internalized and released the miR-221 molecular beacon in the cytoplasm. In the presence of miR-221, the unloaded miR-221 molecular beacon hybridized with the endogenous miR-221, resulting in a strong fluorescence signal (as an imaging agent) and simultaneously miRNA-221 inhibition (as a therapeutic agent), thereby reducing expression of oncogenes [101].

### 5.3. Sgc8 Aptamer-Mediated Drug Delivery

In addition to their ability to differentiate PTK7^+^ T-cell ALL cells from healthy cells, the Sgc8 aptamers can also be engineered as targeting ligands, thereby enabling the selective delivery of therapeutic cargos to target cells. One example is to use the cell targeting Sgc8 aptamers for delivery of anti-tumor chemotherapeutic agents, such as Doxorubicin (Dox) and Daunorubicin, with the aim of reducing off-target toxicity and lowering the concentrations of drug administered, thus reducing potential side effects [102,103]. Sgc8 aptamer-Dox conjugates were therefore designed. In one study, Dox was covalently cross-linked with the 5'-SH group of the Sgc8 aptamers through an acid-liable hydrozone linker [102]. When the Sgc8-Dox conjugate was incubated with a cell mixture, the conjugates recognized target CCRF-CEM cells and efficiently mediated the internalization of Dox through PTK7 receptors. Once inside the cells, Dox was subsequently released into the cytoplasm from the conjugates because of the cleavage of the linker in the acidic endosomal environment. Although the Sgc8 aptamers were attached to Dox, molecular analyses demonstrated that Sgc8-Dox conjugates displayed many of the intrinsic properties of the Sgc8 aptamers, including strong binding strength and high specificity. Moreover, cell viability tests showed that the Sgc8-Dox conjugates were as potent as unconjugated Dox, suggesting that the function of Dox was not affected by aptamer conjugation. Importantly, the Sgc8-Dox conjugates were not internalized by non-target cells; therefore their cellular toxicity was minimal. When combined with different aptamers that specifically target a wide array of cancer cells, this aptamer-drug assembly design lays the foundation for continued development of targeted delivery of a variety of cancers [102].

Another common strategy for drug delivery uses liposomes. Because the cell plasma membrane is made of a dynamic lipid bi-layer, the liposome nanostructure can increase cell permeability and efficiency of drug delivery, akin to the natural process of cell-cell adhesion. One advantage of a liposome-based approach is that the liposomes can be purposefully decorated. To deliver drugs to specific cells, a cell targeting aptamer can be anchored on the liposome to make a cell permeable Sgc8-PEG-liposome nanoparticles [104]. A design in which ~250 copies of Sgc8 aptamers were tethered to a liposome, facilitated multiple aptamer-receptor interactions, provided stronger binding to the target cells and enhanced cellular internalization across the cell membrane [104]. In an initial study, fluorescein-isothiocyanate-dextran was selected as a model molecule and was engineered into a Sgc8 aptamer-coated liposome. Aptamer-conjugated liposomes involving fluorescein-isothiocyanate-dextran were selectively immobilized on the surface membrane of CCRF-CEM cells, but not on non-target NB4 cells [104]. Several studies using specialized lipids, such as diacyl phospholipid and perfluoropentane, to form the liposome nanostructures have also been reported [105].

Hydrogels are water-retainable materials generated from cross-linked polymers, and offer another possibility for aptamer-mediated delivery [106]. Because of their low toxicity and biocompatibility, hydrogel systems have long been recognized as valuable carriers for drug transport. In particular, stimuli-responsive hydrogels that are sensitive to a variety of chemical and physical alternations fulfill many of the critical criteria of targeted drug delivery [106]. For example, an Sgc8 aptamer-incorporated photoresponsive hydrogel system was developed as the recognition unit. The sgc8 aptamers were conjugated to a DNA polyacrylamide chain in the hydrogel systems, which encapsulated Dox and the release of Dox from this system was triggered by exposure to near-infrared light. Flow cytometry indicated that the hydrogels bound to CCRF-CEM cells through the Sgc8 aptamers, then released Dox to kill the cancer cells after exposure to laser light. Collectively, treatment with the Dox-sgc8 aptamer-nanogel system increased death of CCRF-CEM cells from 3% ± 2% to 67% ± 5%, while only 3% ± 2% and 10% ± 3% cell death was observed after incubation with Dox-loaded nanogel conjugated to a DNA library sequence [107].

Finally, akin to liposomes and hydrogels, viral capsids are another nano-material that can serve as a core scaffold for targeted delivery. The protein shell of bacterio-phage MS2 consists of 180 amino acids that are arranged in a 27-nm hollow spherical structure. To functionalize the outer surface of the capsid with targeting capabilities, Sgc8 aptamers were chemically installed through a chemoselective oxidative coupling strategy [108]. Typically, 20 – 40 copies of Sgc8 aptamers were attached on the surface, resulting in a multivalent aptamer-capsid system for targeted delivery. In proof-of-concept studies, acid-liable prodrugs and photodynamic therapy agents have been assembled in the Sgc8 aptamer-conjugated viral capsid core. These agents were able to selectively kill the target cells, while capsids modified with a control DNA strand did not have any cytotoxic effect [109]. This system can be extended to diagnostic imaging by adding radionuclides and contrast agents [109].

### 5.4. Anti-CD30 Aptamer-Mediated siRNA Delivery

CD30 aptamers have been described previously as diagnostic probes for detection of CD30-expressing lymphoma cells. Because of their selectivity and specificity to CD30 receptors, these aptamers were also used as agents to deliver siRNAs to Anaplastic lymphoma kinase (ALK) positive ALCL, which is an aggressive T-cell lymphoma that overexpresses ALK oncogenes and CD30 surface proteins. Through electrostatic interactions, ALK siRNAs and CD30 RNA aptamers were incorporated onto the cationic PEI-citrate carriers to form nanoparticles [110]. Notably, the assembled aptamer-siRNA-nanocomplexes were ~140 nm in diameter such that they are not too small to be rapidly excreted and not too large to be unavailable for cellular uptake. In fact, the nano-particles were stable for more than one day in culture medium and the CD30 aptamers directed the cellular uptake of the siRNA-loaded nanoparticles. Inside the cell, the ALK siRNAs were released from the nanoparticles to specifically silence ALK gene expression, resulting in growth arrest and apoptosis in CD30-expressing ALCL cells [110].

## 6. Conclusions and Perspectives

Aptamers are single stranded non-coding nucleic acids that can be evolved *in vitro* to carry out a specific function by forming a functional three dimensional structure to their targets. Since the first demonstration of SELEX over two decades ago, the use of aptamer technology has shown promise in molecular diagnostics, medical imaging, targeted delivery and pharmaceuticals [111]. Aptamers inhibit enzyme in an extraordinarily strong and specific manner that is comparable and often superior to therapeutic antibodies and small molecules, and yet avoid the toxicity and immunogenicity concerns of these traditional agents [70]. With current advances in cell-based SELEX methodology and versatile chemical modification techniques, aptamers now have remarkable potential to be used as novel delivery vehicles that target a particular cell population, thereby providing enhanced therapeutic efficacy and reduced cellular toxicity [21]. Furthermore, several examples discussed in this review (e.g. Sgc8 aptamer hydrogels) provide complementary approaches for combining the strength of aptamer technology with nanotechnology, offering a versatile platform for disease treatment and diagnosis. However, despite the advances described above, only a few examples have reached extensive clinical development for lymphoma therapy [70,111–115]. Further research in successful translation of aptamer technology should be aimed at the following three main challenges.

First, although the technology for nucleic acid synthesis has improved greatly over the last decade, the cost of industrial-scale production of long, high quality cGMP-grade nucleic acids remains very high and significantly limits clinical development and pre-clinical testing *in vivo* [7,37,116]. Aptamer sequences are usually more than 30 nucleotides long and are extensively modified to protect against nuclease and abrogate type 1 interferon activation, further increasing the cost and difficulties of standard chemical synthesis. Potential solutions to this problem include truncating aptamers to the minimal functional sequence or conjugating multiple aptamers on a polymer [117].

Second, for most uses of aptamer-mediated delivery to date, the aptamer-drug conjugates appear to internalize into the cell through endocytosis pathway [118–120]. Upon internalization, the conjugates encapsulate in endosomal vesicles, then fuse with early endosomes and finally enter into lysosomes, which are the last compartment of the endocytic pathway [121]. Lysosomes are highly acidic (pH ~4.5) and contain various nucleases, which could break down the cargo rapidly [37]. Therefore, efficient escape of therapeutic cargos from the endosome is required and is the most critical challenge in delivery. In this regard, various nanocarriers have been developed to improve the endosomal escape of cargos. Some amine-based materials that have strong buffering capacities, such as PEI, fusogenic lipids, peptides and β-amino ester, have been conjugated to siRNA therapeutics to facilitate endosome escape through the proton sponge effect, which induces rupture of the endosome to release siRNA cargos [122–125]. However, none of these reagents has yet been evaluated in the clinic as a way to promote endosome escape.

Third, the relative paucity of known internalization cell surface receptors limits the wide-applicability of this technique. It is possible that aptamers selected from Cell-SELEX only bind to the cell surface, but do not efficiently internalize into the cytoplasm. In this case, therapeutic cargos escorted by aptamers cannot reach their site of action. Modified cell-internalization SELEX has recently been proposed by Giangrande and colleagues to isolate aptamers that have high affinity and internalization capability. This approach would have a clear advantage compared to selections performed using canonical Cell-SELEX and purified recombinant proteins [126]. In-depth knowledge of cell-surface protein transport and trafficking will also help in identifying new targets for aptamer-mediated delivery.

Continued effort in the development of SELEX technology will enable aptamer-based systems to be developed into their own niches for the treatment and diagnosis of various diseases. In particular, nucleic acids have intrinsic features that can serve as building blocks for bottom-up fabrication of a drug conjugate architecture [127–129]. Their phenomenal diversity in function and versatility in structure make aptamers particularly attractive for many biological applications, and the use of aptamers to deliver cancer treatments, including for lymphoma, is a rapidly growing technique. Aptamers and other therapeutics cargos, including chemotherapeutics and RNA drugs, can assemble into one nanovector that has polyvalent functionalities. Using a similar rationale, we anticipate that novel aptamer-based nanoparticles, such as aptamer-aptamer chimeras or even polyvalent aptamer-siRNA-microRNA nanoparticles, will likely be developed soon to further improve drug delivery and imaging systems. In next few years, aptamer technology is sure to play a critical role in accelerating the translation and development of lymphoma diagnosis and treatment.

## Figures and Tables

**Table 1:** Recent advances in the use of nucleic acid aptamers in lymphoma research.

Aptamers	Targets	Selection strategy	Applications/Status
AS1411 DNA aptamers	Nucleolin	N.A.	Therapeutic agent: Phase IIb clinical trials for treatment of AML (Completed: NCT00512083 and NCT01034410). Targeted delivery for photodynamic drugs, miRNAs and splice-switching oligonucleotides.
NOX-A12 L-RNA aptamers	CXCL12/SDF-1	Spiegelmer technology	Therapeutic agent: Phase IIa clinical trials for the treatment of CLL (on-going: NCT01486797). Diagnostic probes for *in vivo* cancer imaging, microfluidic device and flow cytometry.
Sgc8 DNA aptamers	PTK7	Cell-based SELEX (CCRF-CEM cells)	Targeted delivery for chemotherapy agents and photodynamic drugs using various nanocarriers (e.g. aptamer-drug conjugates, liposomes, hydrogels and viral capsids).
TD05 DNA aptamers	IGHM	Cell-based SELEX (Ramos cells)	Diagnostic probes for biosensor strip and fluorescence imaging. Targeted delivery for photosensitizer agents and micelles.
CD30 RNA aptamers	CD30 receptor	Protein-based SELEX (Recombinant RANK protein)	Diagnostic probes for flow cytometry and immunostaining. Targeted delivery for siRNA-loaded nano-polymers.
